# Maximizing outcomes in penile prosthetic surgery: exploring strategies to prevent and manage infectious and non-infectious complications

**DOI:** 10.1038/s41443-023-00773-7

**Published:** 2023-10-12

**Authors:** Muhammed A. Moukhtar Hammad, David W. Barham, Daniel I. Sanford, Eliad Amini, Lawrence Jenkins, Faysal A. Yafi

**Affiliations:** 1 Department of Urology, University of California, Irvine, Orange, CA USA; 2 USC Institute of Urology and Catherine and Joseph Aresty Department of Urology, Keck School of Medicine, University of Southern California, Los Angeles, CA USA; 3 Artificial Intelligence Center at USC Urology, USC Institute of Urology, University of Southern California, Los Angeles, CA USA; 4 University of California, Irvine, CA USA

**Keywords:** Risk factors, Preventive medicine

## Abstract

Inflatable Penile Prostheses (IPP) implantation is a surgical treatment for patients desiring definitive treatment for erectile dysfunction. While this procedure has proven to be effective, it also carries its own set of unique risks that need to be carefully considered. The article reviews the current understanding of complications associated with penile prosthetic surgery and provides strategies to mitigate these adverse events. This article covers various aspects of IPP implantation, including the risks of infection, bleeding, injury to nearby structures, glans ischemia, and device malfunction. It also discusses the importance of careful preoperative screening to identify risk factors and the implementation of infection reduction strategies such as antimicrobial prophylaxis, skin prep, and operative techniques. In addition, it emphasizes the need for postoperative vigilance and prompt management of any complications that may arise. Overall, the article provides a comprehensive overview of the risks and strategies for mitigating complications associated with IPP implantation. Our recommendations are given based on the current consensus in the field and highlight the importance of careful planning, attention to detail, and effective communication between healthcare providers and patients. Despite the potential risks, this review underscores the fact that complications following penile prosthesis implantation are relatively rare.

## Introduction

Inflatable Penile Prosthesis (IPP) implantation is the gold-standard treatment for erectile dysfunction (ED) [1]. Penile implants, specifically inflatable penile prostheses (IPPs), have been in use since the 1970s and have undergone various improvements over time to enhance patient satisfaction, rigidity, durability, and reduce complications [2]. Penile implant surgery is associated with high satisfaction rates over 80% [3, 4]. As with all surgical interventions however, IPP implantation is not without risks. Complications can include both infectious and non-infectious adverse events such as mechanical malfunction, glans necrosis, and bleeding [5, 6]. In this review, we describe the current understanding of complications in penile prosthetic surgery and discuss strategies to mitigate and manage these adverse events. Although a complete review of all complications of penile implant surgery is beyond the scope of a single review article, we aim to touch on the most common and devastating complications.

## Methodology

A comprehensive search of relevant scientific databases was conducted, including PubMed, MEDLINE, and Scopus, using a combination of keywords related to “penile prosthetic surgery,” “complications,” “infection,” and “outcome.” The selection of topics included in this review was based on the relevance and significance of their impact on outcomes in penile prosthetic surgery.

This review encompasses the most recent publications up until the time of writing, with a focus on the most up-to-date research. It examines relevant articles and studies published within the last 10 years, with a particular emphasis on the most recent 5 years. Thus, it represents the current conventional wisdom on this topic, which has been shaped by the most up-to-date research and expert opinions. A framework was used to guide the selection of topics and the organization of the review article, including sections on risk factors, prevention, and management of infectious and non-infectious complications in penile prosthetic surgery.

## Pre-operative risk assessment and risk reduction

### Diabetes mellitus

Diabetes mellitus is known to increase the risk of postoperative device infection. In 1992, Bishop et al. showed that patients with elevated glycated hemoglobin (HbA1c) had significantly higher rates of infection [7]. Thus diabetes has long been considered a risk factor for implant infection. However, in a systematic review conducted across the Medline database from 1960 to 2014, Christodoulidou and associates found that infection rates among patients with diabetes mellitus have reduced over the years due to device improvement and surgical expertise development [8].

In 2021, Osman et al. reviewed 923 diabetic patients undergoing penile prosthesis placement to determine whether immediate preoperative blood glucose and Hb A1c levels are predictive of postoperative infections in this population [9]. The study included a group of diabetic men who underwent penile prosthesis placement and had their blood glucose and Hb A1c levels measured immediately before surgery. The results of the study showed that there was no significant relationship between immediate preoperative blood glucose and Hb A1c levels and the risk of developing postoperative infections in this group of diabetic men. This suggests that these blood sugar measures may not be useful for predicting the risk of postoperative infections in this specific patient population. However, Lipsky et al. found a significant difference in implant infections when comparing diabetic and nondiabetic patients [10]. Further research is needed to confirm these findings and to explore other potential predictors of postoperative infections in this patient population. In general, diabetes and the associated immunosuppressed state is a concerning factor in regard to implant infection. At our center, we do not have an absolute Hb A1c cutoff, but we do attempt to improve glucose control in diabetic men presenting with poorly controlled diabetes prior to proceeding with penile implant surgery.

### Cardiovascular disease & risk of bleeding

Erectile dysfunction is often a side effect of cardiovascular disease (CVD) [11]. As a result, IPP placement may be a potential treatment in individuals with CVD. IPP is a relatively safe and well-tolerated treatment option for ED in men with CVD and/or risk of bleeding. Patient baseline characteristics from the PROPPER (Prospective Registry of Outcomes with Penile Prosthesis for Erectile Restoration) study were analyzed to determine the most common etiologies before treatment of erectile dysfunction for 1019 patients [12]. CVD was the most common reported condition (31.1%), followed by diabetes (11.8%) and Peyronie’s disease (11.7%). Of those patients receiving an AMS 700, those with CVD and Peyronie’s disease(42.0% and 35.6%, respectively) had significantly less outpatient admissions compared to those treated with radical prostatectomy and those with diabetes (less than 24 h, 56.8% and 52.1%, respectively, *p* < 0.001).

With regards to bleeding risk, Hebert et al. studied men undergoing IPP placement while on anticoagulation with subcutaneous heparin [13]. These men were considered at high risk for postoperative thromboembolic complications necessitating surgery while on heparin. They included 215 men of which 55% were on subcutaneous heparin. Although drain output was higher (99.9 vs 75.6 mL, *p* = 0.001) on postoperative day 0 in those on heparin, there was no difference in the rate of scrotal hematomas (3.8 vs 6.3%, *p* = 0.38) [13].

Patients on antiplatelet and/or anticoagulant (AP/AC) therapy should be closely observed due to the high risk of bleeding associated with penile prosthetic surgery.^13^AUA and International Consultation on Urological Disease (ICUD) published specific guidelines for significant cardiovascular conditions requiring AP/AC use such as congestive heart failure, atrial fibrillation, deep vein thrombosis, coronary stents, mechanical heart valves, and pulmonary embolism [14, 15]. Prosthetic urologists should work closely with the patient’s primary care provider and cardiologist to minimize risks of bleeding and thromboembolic events in men on anticoagulation or antiplatelet therapy.

### Previous abdominal/pelvic surgery

Many patients undergoing penile prosthesis surgery have a history of prior abdominal surgery. Specifically, many men have had a prior radical prostatectomy or cystectomy contributing to the underlying ED, while others may have had other common surgeries such as inguinal hernia repair [16, 17]. All of these procedures can complicate placement of the reservoir which has classically been placed within the retropubic space. Ectopic reservoir placement has been proposed as a solution to men with prior surgeries that may have violated the retropubic space or inguinal canal, which is typically used to gain access to the retropubic space from a penoscrotal approach. Morey popularized the high submuscular placement where the reservoir is placed above the transversalis fascia and below the rectus fascia and muscle [18]. They reported their experience using this technique in 297 consecutive patients [19]. Among these men 100 were randomly selected to undergo a survey. They reported a 1% reservoir herniation rate and 95% patient satisfaction rate. Only 14% of reservoirs were palpable [19]. This group also compared the distance from critical pelvic structures such as the bladder and iliac vessels between reservoirs placed in the retropubic space and those in the high submuscular space. They found reservoirs in the submuscular space were less likely to have mass effect on the bladder or iliac vessels and were 5 times further away from these structures [20].

Kava and colleagues described the trans-fascial placement of the high submuscular reservoir in 2019 [21]. This is a modification of the technique described by Morey, in which the penoscrotal incision is retracted above the inguinal ring and the rectus fascia pierced under direct vision to aid in placement of the reservoir rather than passing the reservoir blindly through the floor of the inguinal canal. They later reported on 107 men who underwent this technique of which there was 1 reservoir herniation, 4 mechanical malfunctions, and 1 patient had autoinflation [22]. In this group, 43 of these patients underwent abdominal imaging which demonstrated correct placement in 79%. Interestingly, they found 7% were intraperitoneal and all of these occurred in men with a history of radical cystectomy. In men with a history of radical cystectomy and neobladder or continent urinary diversion, the authors prefer reservoir placement through a counter-incision.

## Implant infections: incidence, reduction strategies, and management

### Infection incidence

**Fig. 1:**
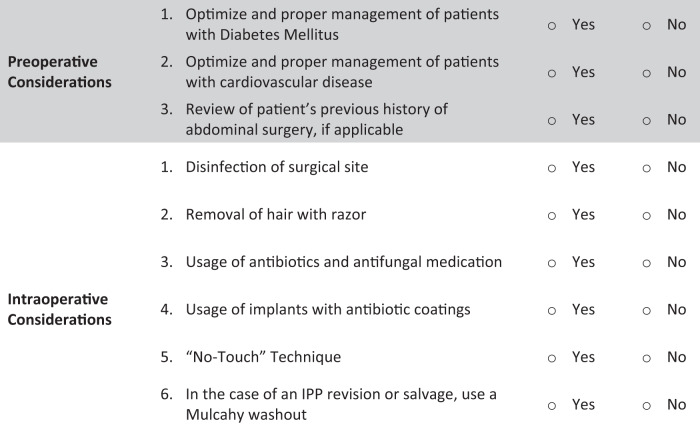
Checklist Summary of Inflatable Penile Prosthesis Placement (IPP) Considerations Preoperatively and Intraoperatively. This checklist outlines crucial preoperative and intraoperative factors to be considered during IPP placement. The checklist serves as a valuable tool for urologists and surgical teams, ensuring a systematic and thorough approach to this complex procedure. Key considerations encompass patient assessment, device selection, surgical technique, and intraoperative troubleshooting. By adhering to this checklist, the team can optimize patient outcomes and minimize complications associated with IPP placement.

Penile prosthesis infection is an infrequent but potentially devastating complication. Infection rates have been shown to vary widely from less than 1% in virgin cases to greater than 10% in revision cases [23, 24]. In a very large, international, multicenter study of 4161 patients undergoing primary penile implant, the incidence of implant infection was found to be 1.1% [25]. In patients with diabetes, Rezaee et al. found the incidence of implant infection to be 3.7% [23]. Further, multiple series have found the infection rate to approach 10% in those undergoing IPP revision surgery [26, 27]. While the rates of infection appear to vary based on specific risk factors, overall infection rates appear to be low. Despite the low infection rates, implant infection is associated with morbidity including need for device explantation, penile length loss, and potential litigious implications [28]. Therefore, prosthetic urologists go to great extents to prevent device infections which are described in the following subsections (Fig. 1).

### Use of chlorhexidine wash

The preoperative use of a chlorhexidine wash is a frequently used method to decrease infection risks. To assess its efficiency in eradicating skin flora, a 2013 prospective, randomized study compared povidone-iodine applied at the surgical site before prosthetic procedure to chlorhexidine. In patients prepared with povidone-iodine, 32% had positive post-preparation cultures while 8% of patients prepared with chlorhexidine had cultures [29]. In a recent study by Karpman et al, they aimed to assess whether dipping sterilized Titan discs in Irrisept solution (0.05% chlorhexidine gluconate) would reduce microorganisms colony counts. Irrisept significantly reduced microbial colony counts from 3 to 6 log_10_ in all species examined when compared to saline [30]. However, since it is an in vitro study, further research will be needed to determine if it has the same effectiveness in vivo.

### Hair removal of surgical site

Complete removal of hair preoperatively is important as the hair may harbor bacteria which could result in postoperative infection. Although many institutes and operating room personnel prefer electric clippers for other surgical procedures, this is a less desirable option for the male genitalia due to the elastic skin of the scrotum. Grober et al. randomized 215 men undergoing surgery of their genitalia to shaving with a razor or clipping with electric razors. They found razors provided more complete removal of hair with less skin trauma. However, they were unable to demonstrate a significant difference in postoperative infection rate [31]. This study may have been underpowered to detect differences in surgical site infection. Their results provide a rationale to support the use of razors. The Sexual Medicine Society of North America gives surgeons freedom to choose either method preoperatively in their guidelines.

### Intraoperative antibiotics and antifungal

Implant infections appear to occur within several months postoperatively. Montgomery et al. found a median time from implant to infection of 2 months (IQR 1–3.3) [32]. Despite the delay in presentation of infection to weeks or months postoperatively, contamination of the field and implant at the time of surgery is the most common source of infection [25]. Historically, skin flora such as *Staphylococcus epidermidis* and *Streptococcus sp*. were the most common pathogenic organisms [33]. These findings were again demonstrated in a 2017 study by Gross et al., where *Staphylococcus sp*. and *Streptococcus sp*. accounted for over 60% of infections [34]. However, they also found a high incidence of *Escherichia coli* (18.2%), anaerobes (10.5%), and fungal species (11.1%). The work by Gross et al. also raised concerns regarding the adequacy of the antibiotic regimens recommended by the AUA and EAU, especially in regard to MRSA, anaerobic, and fungal coverage. Subsequently, the EAU revised its guidelines and now recommends antibiotic prophylaxis; however, they do not recommend specific antibiotic regimens due to a lack of evidence supporting any specific regimen [35]. The AUA currently recommends gentamicin plus a 1^st^/2^nd^ generation cephalosporin or vancomycin [36]. It should be noted that a growing body of literature questions the efficacy of these specific regimens while confirming the benefit of antifungal prophylaxis. A recent, large international study evaluating 4,161 men undergoing primary IPP placement found the use of vancomycin plus gentamicin was associated with a higher risk of infection (HR 2.7, 95% CI: 1.4–5.4, *p* = 0.004) [25]. It also suggested that the use of antifungals decreased the risk of infection by 92% [25].

Several series have similarly demonstrated that approximately 1/3 of infections do not grow any organisms on standard culture [25]. Newer technologies have emerged to isolate bacteria in implant infections. Chung et al. first reported on the use of next-generation sequencing (NGS) in 33 patients undergoing prosthesis explant with 11 of these men having a clinical infection [37]. Of the 11 infected devices, traditional cultures were positive in 100% compared to on 72.7% with NGS [37]. A more recent study by the same group included 83 patients of which only 8 had clinical infections [38]. Among the men with implant infections, *Pseudomonas aeruginosa* was the most common organism identified by NGS [38]. A better understanding of pathogenic organisms will help aid infection prevention.

### Antibiotic coating/impregnated implants

Boston Scientific 700™ series includes: 700 CX, 700 LGX, and the CXR. These three models are covered by Inhibizone™, which is made of minocycline and rifampin to prevent bacterial colonization. Similarly, Coloplast Ltd. produces the Titan and Titan Narrow-Base, which utilizes a covalently bonded hydrophilic coating. This coating can uptake antibiotics when immersed in solution. This allows the surgeon to choose the specific antibiotic coating for Coloplast devices. Additions of antibiotics on both devices have provided a statistically significant reduction in infections compared to devices without this modification [26, 39].

The best choice for antimicrobial solution is generally not agreed upon. In a multicenter study by Towe et al., vancomycin and gentamicin were the most efficacious combination of antibiotics used for dipping a Coloplast device when preventing postoperative infection [40]. Interestingly, rifampin usage as part of the dip was not effective in preventing infection contrary to previous studies which may be attributed to bacterial resistance. Thus, the choice is left to the surgeon’s preference and assessment of each patient’s health status.

### “No-touch” technique

In 2011, Eid introduced the “no-touch” technique for implantation. After initial dissection down to Buck’s fascia, replacement of all instruments and gloves is performed to cover the surgical field with a clear drape. A small window in the drape is created on top of the original incision where the remainder of the procedure is continued. This method minimizes contact with the patient’s skin, surgeon’s hands, surgical instruments, and the implant. This technique decreased infection rate to 0.46% [41].

### Management of implant infections

The standard management of penile prosthesis infection is removal of the infected device with or without salvage. Brant, Ludlow, and Mulcahy first described penile implant salvage in which the infected device was removed, the surgical site was thoroughly irrigated, and a new implant was placed [42]. The irrigation protocol became known as the Mulcahy washout and consisted of kanamycin/bacitracin, half strength hydrogen peroxide, half strength betadine, pressure irrigation with normal saline, vancomycin/gentamicin, and then repeated in reverse order. In this series, 11 men underwent salvage with only 1 repeat infection [42]. Dr. Mulcahy reported his long-term results using this salvage technique in 2000 reporting on 65 patients where he found 82% of patients were free from infection [43]. Over time, this salvage technique has been adopted by many surgeons. After this transition, scrotal complications remained the most common following salvage with an inflatable device. Kohler and colleagues published a pilot series of salvage with a malleable prosthesis in 6 patients. No patients developed infection but 2 underwent conversion from malleable to IPP with another 3 considering conversion to IPP at time of publication [44]. In 2016, Gross et al. published a multicenter study of 58 patients who underwent explantation of an infected IPP with salvage using a malleable prosthesis. They found 93% remained infection free; however, 31% underwent conversion to an IPP at a mean of 6.7 months following salvage surgery [45]. Traditional contraindications to salvage have been necrotic infections, presence of diabetes with purulent drainage, and rapidly developing infections [46].

Due to the high conversion rate seen with salvage using a malleable prosthesis, some surgeons have converted to salvage using an IPP. Jiang et al. recently published a multicenter study of 19 patients with IPP infection who underwent salvage with an IPP [46]. Salvage was performed on average 1.5 days following admission with administration of intravenous antibiotics. Purulence in the operative field was also found in 52.6% of cases. Post-salvage infection occurred in 15.8% of cases [46]. This infection rate is twice as low as the conversion rate to IPP in those salvaged with a malleable. Interestingly, they found purulence in the operative field was not associated with post-salvage infection, which is important as this had previously been considered a contraindication to salvage [46]. Similarly, Chandrapal et al. reported their single-center experience using expanded salvage criteria, including those with purulence or exposed hardware, and found a 92% salvage success rate [47]. Additionally, this group shortened the duration of postoperative oral antibiotics to 2 weeks from the classic 4 weeks [47].

## Postoperative complications

### Device malfunction/revision surgery

Mechanical malfunction is an inevitable complication for IPPs. Early devices were plagued by revision rates as high as 43% at 5 years [48]. Improvements in technology have led to superior device longevity. Recent work has demonstrated median survival over 50% now after 20 years [49, 50]. Given the complex mechanism of the IPP device and the multicomponent design, there are multiple possible points of failure. A malfunctioning device typically results in revision surgery to restore functionality. One dilemma surgeons face at time of mechanical malfunction is whether to revise the malfunctioning component(s) only or replace the entire device. Some advocate for revision which aims to decrease surgical morbidity and costs while others advocate for complete exchange to decrease infection risks associated with biofilm formation on the device.

Campbell et al. evaluated complication rates in 222 men undergoing IPP revision with or without component exchange and found no difference in complication rates between groups [51]. Henry et al. examined 195 patients undergoing IPP revision surgery and found a higher rate of infection and impending erosion in those undergoing revision compared to complete exchange (9.1 vs 5.0%) [52]. A more recent multicenter report of 453 men undergoing IPP revision surgery found a higher rate of infectious (7.1 vs 2.2%) and non-infectious complications (21.2 vs 9.5%) in those undergoing partial component revision compared to complete replacement [53]. Further, this group found a higher rate of complications when the pump and/or cylinders were revised [53]. Therefore, morbidity and complication rates appear lower when the entire device is replaced. However, complete exchange is not without risk, especially in regard to reservoir removal. Major vascular, bladder, and bowel injuries are possible at time of reservoir removal [53]. In a non-infected field, the reservoir can be drained and left in place which has been referred to as the “drain and retain” technique [54].

### Cylinder erosion/impending erosion

The IPP cylinders may erode or threaten to erode into the urethra, the glans, or lateral distal corpora. Erosion is more likely in men with altered penile sensations such as spinal cord injury [55]. Diabetes and vascular disease also appear to increase risk of cylinder erosion or impending erosion [56].

When erosion through the skin or urethra occurs, the device should be explanted and in the case of urethral erosion a urethral catheter should be placed for urinary diversion to facilitate urethral healing [57]. Impending erosion can be managed with extracapsular tunneling which was first described by Clavell-Hernandez in 2021 [58]. Extracapsular tunneling involves making a corporotomy and removing the cylinders. Once the cylinders are removed from the intracapsular space, the posterior aspect of the capsule is incised and the space posterior to the capsule, but within the corpora, is dilated and the new cylinder is placed in this new “extracapsular” space. Clavell-Hernandez originally reported 6 patients treated with extracapsular tunneling for impending erosion, delayed crossover, and hypermobile glans. At a mean follow-up of 6.6 months, all men were engaging in penetrative intercourse without recurrence of the cylinder malposition, malfunction, or implant infection. The authors also routinely perform extracapsular tunneling for cylinder malposition or impending erosion and have been very pleased with the results.

### Glans ischemia

Glans ischemia is a very rare but potentially morbid complication, which can lead to gangrene and even loss of the glans penis. It is a rarely reported complication making it difficult to estimate the true incidence [6]. The largest series of glans necrosis was reported by Wilson et al. including 21 patients [57]. Comorbidities associated with glans necrosis were atherosclerotic CVD (90%), diabetes (81%), smoking (81%), and a history of radiation therapy (48%). Intraoperatively, 67% had a subcoronal incision and 62% had a compressive dressing postoperatively. When glans necrosis is suspected, it is best managed with early explantation. Wilson et al. found 81% of men managed expectantly had loss of a significant amount of glandular tissue, whereas all patients who underwent immediate prosthesis removal healed without tissue loss.

### Reservoir-related complications

Reservoir-related complications are rare, but potentially serious complications associated with IPP placement. The most severe or concerning reservoir related complication is injury to surrounding structures during placement. In a cadaver study, Henry et al. found that the bladder and iliac vessels were 2.6 and 3.3 cm away from the external ring, respectively. When the bladder was drained the distance increased to 6.5 cm [59]. This highlights the importance of draining the bladder prior to reservoir placement in the retropubic space. Overtime reservoir erosion into the bladder has been reported, and a history of pelvic radiation and/or pelvic surgery seem to increase the risk [60]. Bladder injury or reservoir erosion into the bladder typically results in gross hematuria [61]. Axial imaging and/or cystoscopy can be used to diagnose these problems [62]. Vascular injury is quite rare but can involve the iliac vein and branches or the epigastric vessels. If brisk bleeding is noted during reservoir placement then pressure should be held while adequate exposure is obtained. An intraoperative vascular surgery consultation should also be made promptly. Reservoir compression on pelvic vessels has also been reported and may lead to deep vein thrombosis (DVT) and subsequent pulmonary embolism. These cases may require repositioning of the reservoir through a counter incision to alleviate the pathologic compression.

Reservoir autoinflation is a rare but often bothersome problem for patients. This typically arises when an inadequate space is created for the reservoir leading to increased pressure on the reservoir [63]. Continuous autoinflation has also been reported to lead to cylinder erosion due to constant pressure on the distal corpora [64].

Herniation of the reservoir through the inguinal canal has been reported to occur in 0.09–1.4% of patients undergoing IPP placement [65]. Karpman et al. found no significant difference in rates of herniation in reservoirs placed in the retropubic space vs submuscular space [66]. Reservoir herniation can be managed with reservoir repositioning.

## Conclusion

Recent evidence suggests that penile prosthesis implantation can be a safe and effective procedure with favorable outcomes for patients with ED.

Preoperative planning involves a comprehensive assessment of the patient’s medical history, comorbidities, and potential risk factors. It is essential to identify and manage any pre-existing medical conditions, such as diabetes, arteriosclerotic CVD, and other vascular disorders, which may impact the surgical outcomes. Additionally, proper patient selection and counseling are vital to ensure realistic expectations and higher patient satisfaction after the surgery. The team should thoroughly discuss the benefits, potential risks, and possible complications associated with penile prosthesis implantation, enabling patients to make informed decisions. Postoperative care is crucial for ensuring successful outcomes. Proper follow-up and monitoring are essential to detect and manage any post-surgery complications promptly. The management of infections and noninfectious tissue-related issues, such as cylinder erosion or extrusion, requires careful attention and early intervention. Regular communication with the patient and their partner is essential to ensure that they are comfortable with the implant and satisfied with the results. With advancements in surgical techniques and materials, as well as improved perioperative management, IPP’s continue to offer a reliable and effective solution for patients with medication-refractory ED.
